# Closing Editorial for a Special Issue of the *Diagnostics* Journal Titled “Advancements in Interventional Radiology Techniques in Vascular and Extravascular Diseases”

**DOI:** 10.3390/diagnostics16183059

**Published:** 2026-09-21

**Authors:** Francesco Giurazza, Fabio Corvino

**Affiliations:** Vascular and Interventional Radiology Department, Cardarelli Hospital, Via Antonio Cardarelli 9, 80131 Naples, Italy; fabio.corvino@aocardarelli.it

The field of Interventional Radiology (IR) is constantly evolving, driven by the application of modern technologies by physicians who are committed to expanding the boundaries of this fascinating medical branch. In this Special Issue of Diagnostics, titled “Advancements in Interventional Radiology Techniques in Vascular and Extravascular Diseases”, we present a comprehensive range of topics, from oncology [1,2,3,4] to embolization [5,6,7,8,9,10,11] in emergency and elective settings, including also neurovascular diseases [12]. This variety reflects the versatility of IR techniques. We would like to sincerely thank all authors for their invaluable contributions.

It is vital that all manuscripts include detailed technical descriptions of the clinical skills required to properly select and evaluate each patient. This aspect should be included in all IR scientific articles and especially in everyday practice. It is important to note that IR should not be limited to catheters and needles, as is often the case with non-IR colleagues. Instead, the focus should be on proper technical maneuvers, guided by accurate clinical evaluations of patients before and during the procedure. Multidisciplinary consultations are an integral part of the diagnostic and therapeutic pathways for each patient. All interventional radiologists must be able to engage in knowledgeable dialogue regarding clinical evaluations or pharmacological therapies without limiting their role to simply offering advice about the technical feasibility of procedures.

In 2026, two main pillars of IR worldwide will be oncology and embolization. These areas are subject to annual technical developments, largely driven by the medical industry’s support for the development of new devices. From small preliminary studies to multicentric prospective trials, all these elements have resulted in the inclusion of IR in several scientific guidelines, thus certifying its need in almost all medical specialties.

The manuscripts published here illustrate the diverse range of applications of IR.

Locoregional therapies play a vital role in the treatment of both primary and secondary liver cancer diseases, encompassing both endovascular [1,4,9,11] and percutaneous [3] strategies.

Embolization exemplifies perfectly the versatility of IR as demonstrated by the examples provided in this Special Issue, including ectopic pregnancies [2], bleeding [5,6,7], visceral aneurysms [10], and the emerging endovascular musculoskeletal embolization for arthritis [8].

Finally, in the field of neuroradiology, flow diverter stents [12] offer a viable treatment option for life-threatening intracranial aneurysms via peripheral vascular access. This approach significantly reduces recovery time, eliminating the need for complex neurosurgical procedures.

The field of IR is currently attracting substantial interest, and this is set to continue in the future. Evolution and innovation are a constant source of enthusiasm. As physicians, we should express our gratitude to our interventional radiology colleagues. Not only do they provide excellent patient care, but they also dedicate their time to scientific activities, such as reporting on medical literature, clinical experiences, and technical novelties in the medical literature.

## Data Availability

No data required for this type of manuscript.
